# PD 098059, an inhibitor of ERK1 activation, attenuates the in vivo invasiveness of head and neck squamous cell carcinoma

**DOI:** 10.1038/sj.bjc.6690537

**Published:** 1999-07

**Authors:** C Simon, M J Hicks, A J Nemechek, R Mehta, B W O'Malley Jr, H Goepfert, C M Flaitz, D Boyd

**Affiliations:** Departments of Head and Neck Surgery, Cancer Biology and Bioimmunotherapy, The University of Texas MD Anderson Cancer Center, 1515 Holcombe Blvd, Houston, TX, 77030, USA; Department of Otolaryngology – Head and Neck Surgery, Johns Hopkins University, MD, Baltimore, USA; Department of Pathology, Texas Children's Hospital and Baylor College of Medicine, Houston, TX, USA; University of Texas – Dental Branch, Houston, TX, USA

**Keywords:** invasion, MAPK, MMP-9, PD 098059

## Abstract

Increased mortality of patients with oral cancer largely reflects the local and regional spread of the disease. The invasiveness of these tumours requires hydrolases which are regulated through AP-1-dependent transcriptional mechanisms. Since the amount/activity of transcription factors bound to the AP-1 motif are regulated partly through the extracellular signal-regulated kinases (ERK1/ERK2), we determined the effect of PD 098059, an inhibitor of ERK1/ERK2 activation, on the in vivo invasiveness of a human squamous cell carcinoma cell line (UM-SCC-1) derived from the oral cavity. We utilized the floor of mouth musculature consisting of the mylohyoid, geniohyoid and genioglossus muscle (which are sequentially arranged), as a natural barrier to assess tumour spread in vivo in the nude mouse. Mice were inoculated with tumour cells superficial to the mylohyoid muscle. After 18 days, tumours were injected with either empty liposomes (control) or liposomes containing 5 μM PD 098059 and, after an additional 22 days, the jaws of mice examined histologically. Highly infiltrative tumours, which had penetrated the genioglossus muscle, were evident in 10/12 control mice. In contrast, in 9/12 mice in which the tumours were injected with PD 098059, tumours did not extend beyond the mylohyoid or geniohyoid muscles. Tumours penetrated bone nutrient canals in 7/12 control mice but in only 3/12 PD 098059-treated mice. Neurotropism, characteristic of aggressive oral squamous cell carcinoma, was evident in 6/12 control mice but was completely abolished (0/12 mice) in the PD 098059-treated mice. Using a staging system based on the muscle layer involved, neurotropism, as well as bone involvement, we found the inhibition of invasion to be statistically significant (*P* < 0.01). The reduced invasiveness of the PD 098059-liposome-treated oral cancers was associated with diminished 92-kDa type IV collagenase and ERK1/ERK2 activities but was not a consequence of a slower tumour growth rate. This is the first study to demonstrate reduced in vivo invasiveness of a malignancy brought about by an inhibitor of ERK1/ERK2 activation. These results raise the exciting possibility that second generation PD 098059 congeners may reduce the spread of the disease in patients afflicted with oral cancers. © 1999 Cancer Research Campaign


*British Journal of Cancer* (1999) **80**(9), 1412–1419
© 1999 Cancer Research Campaign
Article no. bjoc.1998.0537

### PD 098059, an inhibitor of ERK1 activation, attenuates the in vivo invasiveness of head and neck squamous cell carcinoma

**C Simon[1,*], MJ Hicks[5], AJ Nemechek[1], R Mehta[3], BW O'Malley Jr[4], H Goepfert[1], CM Flaitz[6] and D Boyd[2]**

Departments of [1]Head and Neck Surgery, [2]Cancer Biology and [3]Bioimmunotherapy, The University of Texas MD Anderson Cancer Center, 1515 Holcombe Blvd, Houston, TX 77030, USA; [4]Department of Otolaryngology – Head and Neck Surgery, Johns Hopkins University, Baltimore, MD, USA; [5]Department of Pathology, Texas Children's Hospital and Baylor College of Medicine, Houston, TX, USA; [6]University of Texas – Dental Branch, Houston, TX, USA

**Summary** Increased mortality of patients with oral cancer largely reflects the local and regional spread of the disease. The invasiveness of these tumours requires hydrolases which are regulated through AP-1-dependent transcriptional mechanisms. Since the amount/activity of transcription factors bound to the AP-1 motif are regulated partly through the extracellular signal-regulated kinases (ERK1/ERK2), we determined the effect of PD 098059, an inhibitor of ERK1/ERK2 activation, on the in vivo invasiveness of a human squamous cell carcinoma cell line (UM-SCC-1) derived from the oral cavity. We utilized the floor of mouth musculature consisting of the mylohyoid, geniohyoid and genioglossus muscle (which are sequentially arranged), as a natural barrier to assess tumour spread in vivo in the nude mouse. Mice were inoculated with tumour cells superficial to the mylohyoid muscle. After 18 days, tumours were injected with either empty liposomes (control) or liposomes containing 5 μM PD 098059 and, after an additional 22 days, the jaws of mice examined histologically. Highly infiltrative tumours, which had penetrated the genioglossus muscle, were evident in 10/12 control mice. In contrast, in 9/12 mice in which the tumours were injected with PD 098059, tumours did not extend beyond the mylohyoid or geniohyoid muscles. Tumours penetrated bone nutrient canals in 7/12 control mice but in only 3/12 PD 098059-treated mice. Neurotropism, characteristic of aggressive oral squamous cell carcinoma, was evident in 6/12 control mice but was completely abolished (0/12 mice) in the PD 098059-treated mice. Using a staging system based on the muscle layer involved, neurotropism, as well as bone involvement, we found the inhibition of invasion to be statistically significant ($P < 0.01$). The reduced invasiveness of the PD 098059-liposome-treated oral cancers was associated with diminished 92-kDa type IV collagenase and ERK1/ERK2 activities but was not a consequence of a slower tumour growth rate. This is the first study to demonstrate reduced in vivo invasiveness of a malignancy brought about by an inhibitor of ERK1/ERK2 activation. These results raise the exciting possibility that second generation PD 098059 congeners may reduce the spread of the disease in patients afflicted with oral cancers.

**Keywords:** invasion; MAPK; MMP-9; PD 098059

The high mortality rate of patients with advanced oral cancer most often reflects the local and regional spread of the disease. Thus, invasion of soft tissue can lead to the destruction of vital structures, such as major nerves and the carotid artery, and to overwhelming bacterial infection, the latter culminating in uncontrolled systemic infection. Additionally, and depending on the site of tumour origin, invasion of important structures such as the larynx may cause significant morbidity affecting quality of life.

Tumour cell infiltration into these surrounding structures requires the hydrolysis of extracellular matrix components, such as type IV collagen, laminin, fibronectin and heparan sulphates (Tryggvason et al, 1987). This extracellular matrix hydrolysis is largely brought about by the activity of proteases, including the matrix metalloproteases (MMPs) (Matrisian, 1992), urokinase-type plasminogen activator (u-PA) (Ossowski et al, 1983; Axelrod et al, 1989; Wilhelm et al, 1995), endoglycosidases (Nakajima et al, 1992), and cathepsins (Rempel et al, 1994).

In efforts to reduce tumour invasiveness, several attempts have been made to block the activity of these proteases with specific synthetic inhibitors directed at a particular enzyme (Anderson et al, 1996; Xing et al, 1997). However, the disadvantage of targeting one protease is that the activity of other enzymes that contribute to tumour invasiveness is unaffected. Considering this limitation, we have argued that an agent with the potential to coordinately repress the synthesis of multiple proteases (Gum et al, 1997; Lengyel et al, 1997) may represent a more effective way of controlling tumour cell invasiveness. How may this be achieved? We propose a line of attack that is aimed at AP-1-dependent transcriptional regulation since a number of proteases, including the 92-kDa type IV collagenase (MMP-9) (Sato and Seiki, 1993), urokinase, stromelysin-2 and type I collagenase, which are all expressed in oral squamous cell carcinoma (Clayman et al, 1993; Muller et al, 1993), are controlled via AP-1 motifs in their promoters (Auble and Brinckerhoff, 1989; Sirum and Brinckerhoff, 1989; Nerlov et al, 1991; Sato and Seiki, 1993). These motifs are bound with transcription factors that are regulated, in turn, partly through the extracellular signal-regulated kinase (ERK) subset of mitogen-activated

*Received 8 July 1998*
*Revised 4 November 1998*
*Accepted 9 November 1998*

*Correspondence to:* D Boyd

*Present address: Department of Otolaryngology – Head and Neck Surgery, University of Tuebingen, Germany.
C Simon and MJ Hicks share first co-authorship.

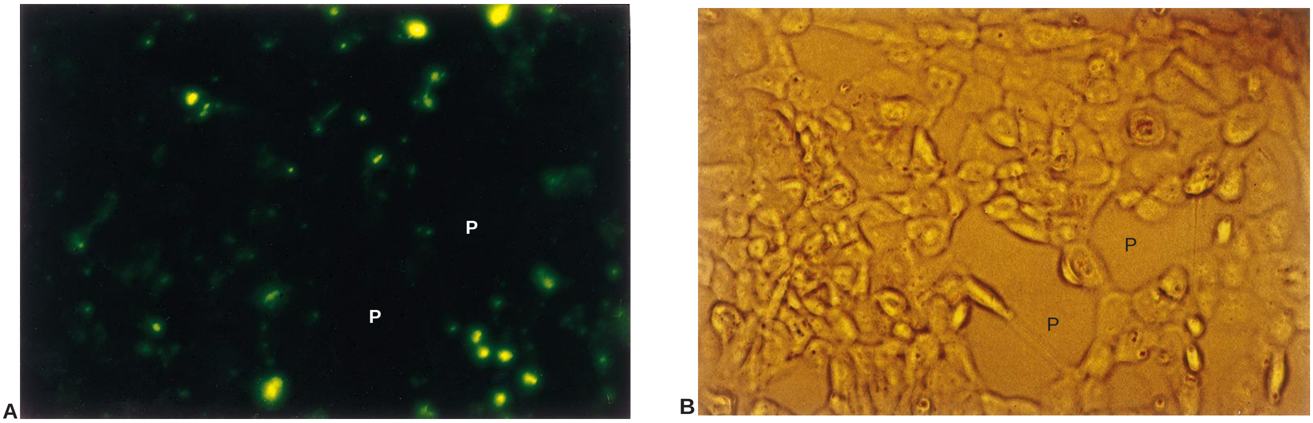

**Figure 1** Delivery of PD 098059 into the cells with neutral phospholipid liposomes. UM-SCC-1 cells were grown to 95% confluence and treated with culture medium containing 200 µg ml$^{-1}$ of fluorescent-labelled liposomes. Liposome uptake into the cells was visualized using an inverted fluorescence microscope Nikon Diaphot using a blue filter (**A**). The monolayered cells were visualized by phase contrast (**B**)

protein kinases (MAPK) (Gille et al, 1992; Frost et al, 1994). The ERKs are the terminal kinases in a signal transduction pathway connecting cell surface growth factor/cytokine receptors to the nuclear transcriptional machinery. Following growth factor/cytokine receptor activation, Ras, c-Raf-1, and MEK-1 are sequentially activated by phosphorylation (Kyriakis et al, 1992; Huang et al, 1993; Jelinek et al, 1994; Zheng and Guan, 1994) leading to altered transcription factor activity and/or synthesis (Pulverer et al, 1991; Gille et al, 1992; Bogoyevitch et al, 1995; Abdel-Hafiz et al, 1996; Janknecht, 1996). Indeed, we previously demonstrated that the elevated expression of MMP-9 and urokinase, two enzymes shown to be involved in head and neck squamous cell carcinoma (HNSCC) invasion (Ossowski, 1992; Clayman et al, 1993; Juarez et al, 1993) was driven, at least in part, through a constitutively activated ERK1 (Simon et al, 1996; Gum et al, 1997; Lengyel et al, 1997). Moreover, the specific inhibition of MEK-1, an immediate upstream activator of ERK1 by PD 098059 ([2-(2′-amino-3′ methoxyphenyl)-oxanaphthalen-4-one] (Alessi et al, 1995; Dudley et al, 1995) substantially reduced both MMP-9 and urokinase expression in cultured squamous cell carcinoma of the oral cavity (Simon et al, 1996; Gum et al, 1997).

Accordingly, we undertook a study to determine the ability of this specific inhibitor of ERK1 activation to attenuate the invasiveness of an oral cancer cell line orthotopically implanted into the floor of mouth of nude mice. We demonstrate that PD 098059 can, when delivered via dipalmitoyl phosphatidylcholine (DPPC) liposomes as a carrier, diminish the invasiveness and abolish the perineural invasion of tumours derived from UM-SCC-1 cells. This attenuation in invasion was associated with reductions in MMP-9 and ERK activities in the tumours.

### MATERIALS AND METHODS

### Tissue culture

UM-SCC-1 is a squamous cell carcinoma cell line originally derived from an oral cancer (obtained from Dr Thomas Carey, University of Michigan, Ann Arbor, MI, USA). UM-SCC-1 cells were maintained in McCoy's 5A culture medium supplemented with 10% fetal bovine serum (FBS). For the collection of conditioned medium for zymography, 80% confluent UM-SCC-1 cells were incubated in serum-free medium (McCoy's 5A medium supplemented with 5 µg ml$^{-1}$ insulin, 10 ng ml$^{-1}$ epidermal growth factor and 4 µg ml$^{-1}$ transferrin) for 48 h with various amounts of PD 098059 (kindly provided by Dr Alan Saltiel, Parke Davis, Ann Arbor, MI, USA) entrapped in liposomes or carrier alone (liposomes). The culture medium was collected and relative cell number determined using the metabolism of the 3-(4,5-dimethylthiazol-2-yl)-2,5-diphenyltetrazolium bromide (MTT) vital stain (Mossman, 1983).

### Tumour tissue preparation

Tumour tissue extracts for gelatin-zymography and in-gel kinase assay were prepared in buffer A (1% NP40 (octylphenoxy polyethoxy ethanol), 25 mM Tris–HCl (pH 7.4), 25 mM sodium chloride, 1 mM sodium vanadate, 10 mM NaF, 10 mM sodium pyrophosphate, 10 nM okadaic acid, 0.5 mM EGTA, and 1 mM phenylmethylsulfonyl fluoride) after snap freezing in liquid nitrogen and homogenization.

### Preparation of liposomal PD 098059

Multilammellar vesicles containing PD 098059 were prepared as described elsewhere (Mehta, 1996) by solubilizing dipalmitoyl phosphatidylcholine (DPPC) (Avanti Polar-Lipids Inc., Alabaster, AL, USA) and drug (9:1 molar ratio) in 80% tertiary butanol. The drug-lipid solution was then sonicated, frozen and lyophilized for 48 h. The lyophilized powder was stored at –20°C until use. Liposomes were prepared by reconstituting the lyophilized powder in sterile saline solution, the suspension centrifuged at 48 000 ***g*** for 60 min, and the pellet resuspended in normal saline. The concentration of encapsulated drug was determined spectrophotometrically at 294 nm after solubilization of an aliquot of the liposomes with methanol.

For qualitative determination of the uptake of liposomes into cells, fluorescent-labelled liposomes were prepared by solubilizing DPPC and palmitoyl-(nitro-benzoxadiazol-amino-dodecanoyl)-phosphatidylcholine (P-NBD-PC) (10:0.1 molar ratio) in 80% tertiary butanol and reconstituted in saline solution. UM-SCC-1 cells were grown to 95% confluence and treated with culture medium containing 200 µg ml$^{-1}$ of fluorescent-labelled liposomes for 24 h. Liposome uptake into the cells was visualized using an inverted fluorescence microscope Nikon Diaphot (Nikon Inc, Garden City, NY, USA) using a blue filter.

*© 1999 Cancer Research Campaign*

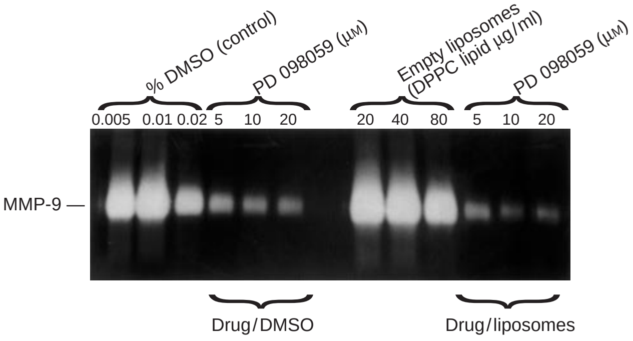

**Figure 2** Reduction of MMP-9 secretion in PD 098059-treated UM-SCC-1 cells grown in vitro. UM-SCC-1 cells were treated for 48 h with the indicated agent and/or carrier. After this time, conditioned medium was harvested and centrifuged to remove the liposomes. Aliquots of conditioned medium normalized for differences in cell number were analysed by zymography

### Animal experiments and statistical analysis

The establishment of tumours in the floor of mouth of nude mice was achieved essentially as described elsewhere (O'Malley et al, 1995). Briefly, athymic female nude mice (*nu/nu*, Harlan Sprague-Dawley), aged 6–7 weeks, were anaesthetized by intraperitoneal injection of ketamine/xylazine (10 and 1 mg $ml^{-1}$ respectively). Using a 250 μl syringe (Hamilton, Reno, NV, USA) and 30-gauge needle, a 50 μl solution containing $5 \times 10^6$ human UM-SCC-1 squamous cells in Hank's buffered saline was injected into the subcutaneous tissue superficial to the mylohyoid muscle of the nude mice. To prevent inadvertent needle placement into the musculature, mice were placed in the supine position, the cervical overlying skin placed on tension, the needle oriented parallel to the floor of mouth musculature and the tumour innoculum delivered into the subcutaneous tissue. The needle was removed with no grossly detectable leakage. The mice were maintained in standard animal housing for 43 days. No antibiotics or supplemental pain control measures were employed. The animals were monitored daily.

For the injection of liposomes with, and without, PD 098059 the nude mice were anaesthetized as described above. After measurement of gross tumour size in three dimensions, the tumour was injected (30-gauge needle) with drug/liposome at three separate sites (equidistant of each other) using a total volume corresponding to one-quarter of the tumour volume. At this point, tumours were 75–100 $mm^3$. Treatment was started 18 days after mice were inoculated with tumour cells. Tumours subsequently received drug/liposome injections on day 23, 28 and every third day thereafter until day 40. Mice were then sacrificed by carbon dioxide inhalation. The treated mice showed no change in eating or other behaviour habits over the course of the treatment. For histopathological evaluation of invasion, animal jaws were fixed in 10% buffered formalin, decalcified, routinely processed and embedded in paraffin. Serial 3-μM sections were cut and stained with haematoxylin and eosin. Histological sections were examined by two individuals simultaneously, both blinded to the particular treatments of each animal. The difference in invasion between PD 098059-treated and untreated groups were tested for statistical significance using Fisher's exact test.

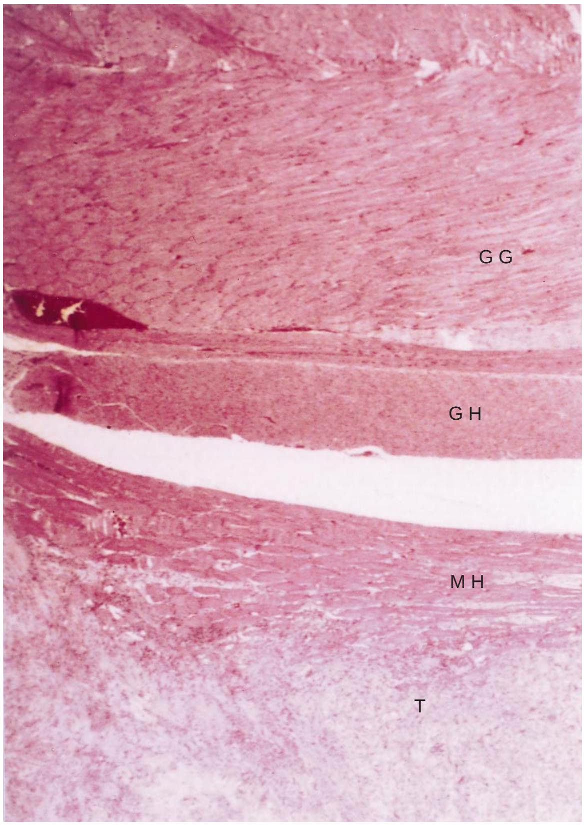

**Figure 3** Establishment of tumours in the floor of mouth. UM-SCC-1 cells were injected into the subcutaneous tissue superficial to the mylohyoid muscle. After 18 days, mice were sacrificed and animal jaws fixed in 10% buffered formalin. Tissue sections were stained with haematoxylin and eosin. T, tumour; MH, mylohyoid muscle; GH, geniohyoid muscle; GG, genioglossus muscle. Original magnification = 40×

### Zymography on conditioned medium and tumour tissues

Zymography was performed essentially as described previously (Gum et al, 1996) using polyacrylamide gels containing 0.1% (w/v) gelatin. After staining for protein with Coomassie brilliant blue, gelatinolysis was detected as white zones in a dark field.

### In-gel kinase assays for ERK activity and Western blotting

Kinase assays were performed as described by us (Lengyel et al, 1997). Briefly, cells were extracted with buffer A (see Tumour Tissue Preparation). Extracted protein was either immunoprecipitated with 2 μg of the anti-ERK1/ERK2 antibody (Santa Cruz Biotechnology # 93, Santa Cruz, CA, USA) and 50 μg of protein A agarose or resolved by sodium dodecyl sulphate poliacrylamide gel electrophoresis (SDS-PAGE) and transferred to a nitrocellulose filter for Western blotting. For kinase assays, the pellet was washed with buffer A and resuspended in 2× sample buffer, and the immune complexes were electrophoresed in a polyacrylamide gel containing myelin basic protein. The gel was treated sequentially with buffers containing 20% 2-propanol, 5 mM 2-mercaptoethanol, 6 M guanidine hydrochloric acid and 0.04% Tween-40–5 mM 2-mercaptoethanol. The gel was then incubated at 25°C for 1 h with 10 μM adenosine 5′-triphosphate (ATP) and 25 μCi of [($^{32}$P]ATP in a buffer containing 2 mM dithiothreitol–0.1 mM EGTA–5 mM

 © 1999 Cancer Research Campaign magnesium chloride, washed in a solution containing 5% trichloroacetic acid and 1% sodium pyrophosphate, dried, and autoradiographed. For Western blotting the membrane was blocked with 3% bovine serum albumin before incubation with primary antibody and horseradish peroxidase-conjugated anti-rabbit IgG. Immunoreactive bands were visualized by enhanced chemiluminescence (ECL) (Amersham, Arlington Heights, IL, USA).

### RESULTS

### DPPC liposomes efficiently deliver PD 098059 into UM-SCC-1 cells

PD 098059, a specific inhibitor of MEK-1 (Alessi et al, 1995; Dudley et al, 1995), is a lipophilic compound and therefore requires a carrier for in vivo delivery. Thus, in order to deliver the drug intratumourally, we elected to use liposomes as a vehicle for PD 098059. We first determined the ability of liposomes to deliver PD 098059 into cultured squamous cell carcinoma cells. Fluorescence-labelled liposomes formulated from the phospholipid DPPC efficiently coalesced (Figure 1 A, B) with the surface membranes of cultured UM-SCC-1 squamous cell carcinoma cells, the cell line intended for use in the in vivo invasion model. Note the absence of fluorescence from the exposed tissue culture plate (P). To confirm that the liposome-delivered PD 098059 was able to suppress MMP-9 synthesis, UM-SCC-1 cells were assayed for gelatinase activity by zymography. A gelatinase activity, indistinguishable in size from MMP-9 (92 kDa), was detected in the conditioned medium from cultured UM-SCC-1 cells (Figure 2). This activity was unaltered by increasing the amount of empty liposomes. Conversely, the intensity of the MMP-9 activity was decreased with increasing amount of liposome-delivered PD 098059. In fact, the decrease brought about by way of liposome-entrapped PD 098059 was quantitatively similar to that achieved with equivalent concentrations of PD 098059 solubilized in dimethyl sulphoxide (DMSO).

### PD 098059 attenuates in vivo invasion and abolishes perineural invasion by tumours formed from UM-SCC-1 cells

To study the effect of PD 098059 on tumour invasion in vivo we first developed a model that allowed us to quantitate the invasiveness of tumours formed from UM-SCC-1 cells. This made use of the floor of mouth musculature in which the mylohyoid (most superficial), geniohyoid and genioglossus (deepest) muscles are found sequentially. Towards this end, we orthotopically implanted $5 \times 10^6$ UM-SCC-1 squamous cell carcinoma cells transcervically into the subcutaneous tissue to a depth superficial to the mylohyoid muscle in the floor of mouth of the nude mice. The different muscle layers (mylohyoid – MH, geniohyoid – GH, genioglossus – GG), which overlie each other, in the floor of mouth of the nude mouse served as natural invasion barriers (Figure 3). A staging system was devised based on muscle layers invaded and the involvement of blood vessels, bone, nerves and lymph nodes (Table 1) to allow the separation of tumours into low (stage I) and high (stage II) invasiveness.

We then injected the drug-liposome intratumourally and compared the extent of invasion between treatment and control groups. To exclude a potentially inhibitory effect of the drug on tumour establishment, mice were not treated for the first 18 days to allow tumour formation and invasion of the adjacent mylohyoid muscle by the tumour (T) cells (Figure 3). Then, mice were randomized into a treatment (5 μM PD 098059-liposomes, 12 mice) and a control group (empty liposomes, 12 mice) and treated for the next 22 days. After this time, mice were then sacrificed and the floor of mouth excised, stained and examined histologically. We found that tumours which were treated with empty liposomes were highly infiltrative (Figure 4A) with the malignant cells (T) penetrating (arrowhead) into the genioglossus muscle (GG). Lower magnification (Figure 4B) clearly indicates the tumour front (arrowhead) infiltrating the genioglossus muscle (GG) with the lingual mucosa (LM) (superficial aspect of the tongue) clearly evident. In order for this muscle layer to be involved, tumour cells must traverse both the mylohyoid and the geniohyoid muscles which underlie the genioglossus. Tumour cells (T) treated with empty liposomes also invaded the vascular space (V) of blood vessels (Figure 5A) and the nutrient canals of bone (B) (Figure 5B). UM-SCC-1-formed tumours demonstrated neurotropism (Figure 5C) as evident by the presence of tumour cells (T) within the perineural space. Neurotropism is characteristic of aggressive head and neck squamous cell carcinomas in man and is associated with a poor outcome for patients (Carter et al, 1983; Goepfert et al, 1984).

These findings were in sharp contrast to the results with the treatment group. Whereas most mice (10/12, 83%) treated with empty liposomes had highly infiltrative tumours involving the genioglossus muscle, the majority of mice (9/12 mice (75%)) treated with PD 098059-liposomes had tumours that penetrated, but did not extend beyond, the mylohyoid (MH) or geniohyoid

**Table 1** Histological criteria defining minimally (stage I) and highly (stage II) invasive tumours

| Stage | |
|---|---|
| I | II |
| Tumour involves mylohyoid or geniohyoid muscle with, or without, vascular invasion | Tumour invades genioglossus muscle with, or without, bone, vascular invasion<br>*or*<br>Invasion of any muscle with perineural invasion or lymph node involvement |

**Table 2** Treatment of tumours with PD 098059-liposomes reduces their invasiveness

| | No. of mice | |
|---|---|---|
| | Stage I | Stage II |
| Liposome only (day 40) | 1 | 11 |
| PD 098059-liposome (day 40) | 9 | 3 |

UM-SCC-1 cells were injected into the subcutaneous tissue superficial to the mylohyoid muscle. After 18 days, mice were treated with empty liposomes (12 mice) or PD 098059-liposomes (12 mice). Tumours subsequently received liposome only or drug-liposome injections on day 23, 28 and every third day thereafter until day 40. At that point, all mice were sacrificed and animal jaws fixed in 10% buffered formalin. Tissue sections were stained with haematoxylin and eosin. Using the histological criteria described in Table 1, the number of mice with minimally (stage I) or highly (stage II) invasive tumours was enumerated.

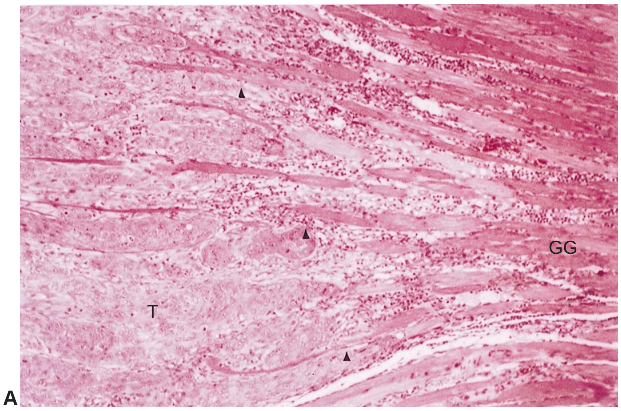

**Figure 4** Tumours treated with empty liposomes invade the genioglossus muscle. UM-SCC-1 cells were injected into the subcutaneous tissue superficial to the mylohyoid muscle. After 18 days, mice were treated with empty liposomes. After an additional 22 days, mice were sacrificed and animal jaws fixed in 10% buffered formalin. Tissue sections were stained with haematoxylin and eosin. (**A**) Original magnification = 100×. (**B**) Original magnification = 40×. T, tumour; GG, genioglossus muscle; arrowhead, infiltrating tumour cells; LM, lingual mucosa

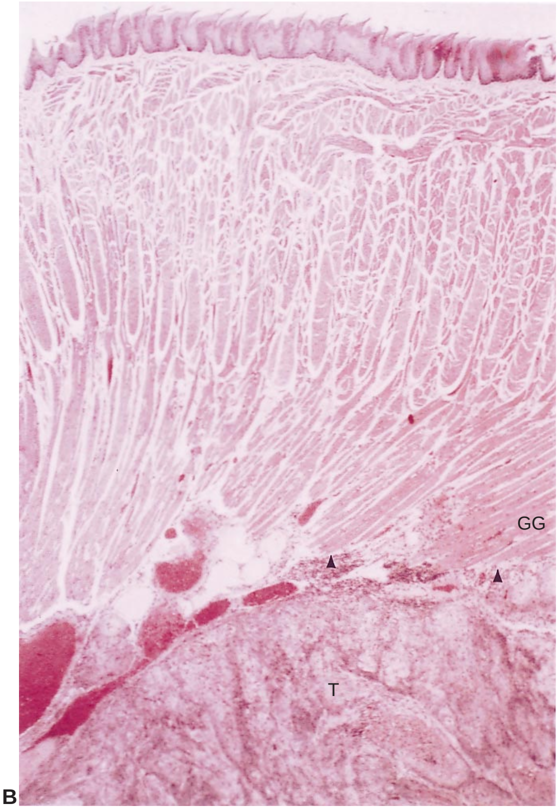

(GH) muscles (Figure 6). Tumours treated with empty liposomes also invaded bone (7/12 mice, 58%) while this was apparent in only 3/12 (25%) mice given PD 098059-liposomes. Remarkably, perineural invasion, a hallmark of aggressive head and neck cancer (Carter et al, 1983; Geopfert et al, 1984), and apparent in 6/12 mice (50%) in the empty liposome treatment group was completely abrogated (0/12) in the PD 098059-treated group ($P < 0.05$) While 11 of 12 mice receiving liposomes only were determined to have stage II (highly invasive) tumours, only three of 12 mice treated with PD 098059-liposomes were found with stage II tumours (Table 2). As a corollary, while one of 12 mice receiving liposome alone had minimally invasive (stage I) tumours, the majority (nine of 12 animals) receiving the MEK1 inhibitor were classified as having stage I tumours (Table 2). These differences were statistically significant (Fisher-exact test, $P < 0.01$).

It is unlikely that the observed effects brought about by PD 098059 reflect an inhibition of proliferative activity in the treated tumours, since the mitotic index was not found to be significantly different as compared to tumours in the control group (median 24 ± 13.3 and 23 ± 6.1 respectively). Further, we found a poor relationship (Student's *t*-test, $P = 0.31$) between invasion stage and tumour volume.

### Treatment of UM-SCC-1-derived tumours in vivo with PD 098059 reduces MMP-9 and ERK1 activity

Presumably the reduced in vivo invasiveness brought about by PD 098059 reflects, at least in part, diminished proteolysis. To address this possibility, we analysed control and treated tumours for MMP-9 activity. Tumours harvested from untreated mice contained a gelatinase activity, which was indistinguishable in size (92-kDa) from MMP-9. However, the intensity of this activity was substantially reduced in tumours treated with the MEK1 inhibitor (Figure 7A). In contrast, we did not observe a reduction in the amount of a gelatinase activity, which was indistinguishable in size (72-kDa) from MMP-2.

To confirm that the PD 098059 in fact reduced ERK1 activity, residual tumour tissue from histological and protease determinations was homogenized. The resulting tumour extract was then incubated with an anti-ERK1/ERK2 antibody and the immunoprecipitated material subjected to an in-gel kinase assay. ERK1 activity was reduced in a PD 098059-treated tumour compared with a control tumour that had received only empty liposomes (Figure 7B). The reduced ERK1 activity was not a consequence of a smaller amount of the MAPK protein as shown by Western blotting (Figure 7C). Taken together, these data suggest that the prevention of ERK activation by the MEK inhibitor PD 098059 reduces tumour cell invasion in vivo possibly as a consequence of reducing the expression of the metalloproteinase MMP-9.

### DISCUSSION

The local and regional spread of head and neck squamous cell carcinomas is most often life-threatening to patients afflicted with this malignancy. Thus, there is an urgent need to control the ability of the tumour cells to spread locally and regionally. Although tumour invasion is a complex process, degradation of the surrounding extracellular matrix is a prerequisite step and accomplished largely via proteolysis (Tryggvason et al, 1987; Wilhelm et al, 1989). Accordingly, a reduction in protease synthesis would be expected to attenuate tumour invasiveness. However, achieving this goal is complicated by two issues; firstly that multiple enzymes are

 © 1999 Cancer Research Campaign

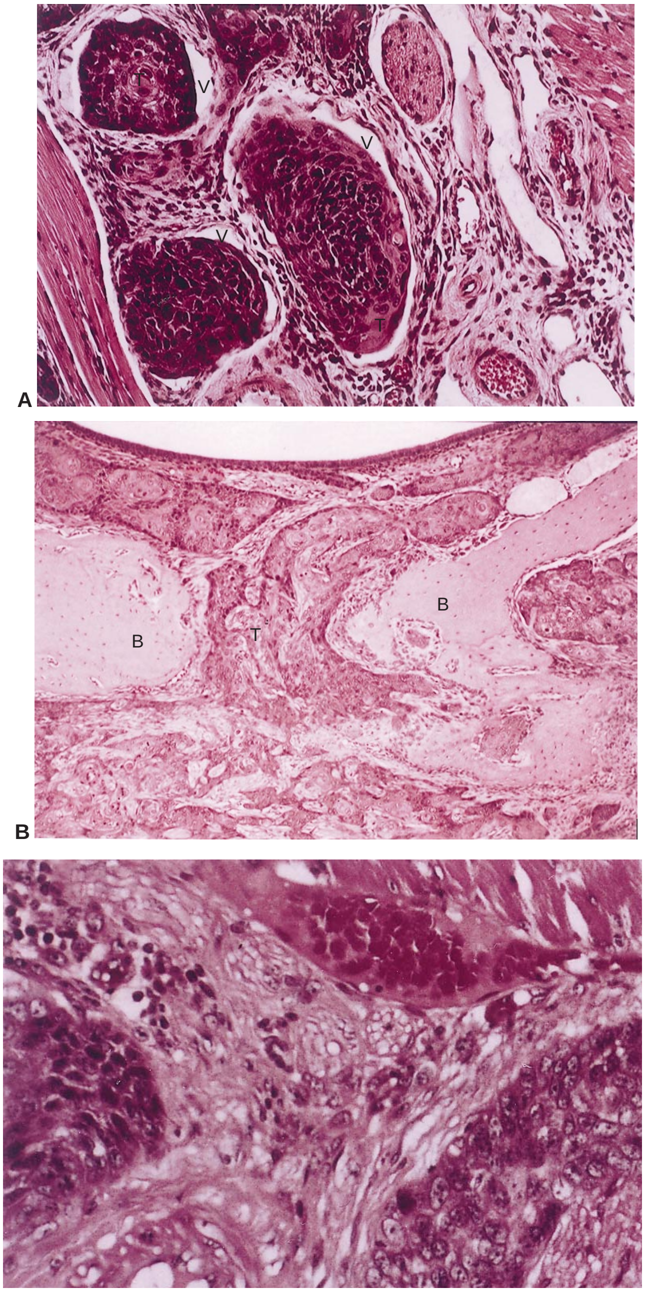

**Figure 5** Tumours treated with empty liposomes invade the vascular space, bone and nerves. Tumour generation, tissue processing and staining were as described in the legend to Figure 4. V, vascular space; T, tumour; B, bone; N, nerve. (**A**) Original magnification = 200×. (**B**) Original magnification = 100×. (**C**) Original magnification = 200×

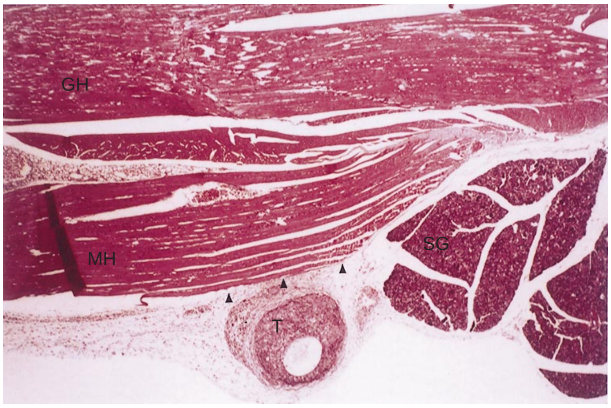

**Figure 6** Reduced invasiveness of tumours treated with PD 098059-liposomes. Tumour generation, tissue processing and staining were as described in the legend to Figure 4. MH, mylohyoid muscle; GH, geniohyoid muscle; SG, salivary gland; T, tumour. Original magnification = 40×

required for invasiveness (Ossowski, 1992), and second that the regulation of expression of these hydrolases is brought about by the concerted action of divergent mitogens such as transforming growth factor-α (Jensen and Rodect, 1993; Lyons et al, 1993) and hepatocyte growth factor (Pepper et al, 1992). Thus, the targeting of any one hydrolase or any one mitogen may prove inefficient in repressing tumour invasiveness. We explored instead the potential of antagonizing a signalling cascade utilized by multiple growth factors for 'driving' the expression of divergent proteases. Several hydrolases (MMP-9, urokinase, stromelysin-2, type I collagenase) (Sirum and Brinckerhoff, 1989; Auble and Brinckerhoff, 1991; Nerlov et al, 1991; Sato and Seiki, 1993) implicated in the spread of oral squamous cell carcinoma are AP-1-regulated, and since the amount and/or activity of transcription factors bound to this motif is regulated through ERK1 (Pulverer et al, 1991; Gille et al, 1992; Agarwal et al, 1995; Bogoyevitch et al, 1995), we undertook a study to determine if an inhibitor (PD 098059) of this MAPK attenuated the invasiveness of orthotopically-implanted tumours established from UM-SCC-1 cells. We report for the first time a dramatic anti-invasive effect of this agent in vivo. Equally important, treatment of tumours with PD 098059 completely abrogated neurotropism. This is an important observation since head and neck cancer patients with perineural invasion have a high incidence of local recurrence (47%) and metastasis (35%) (Geopfert et al, 1984; Rowe et al, 1992).

Although there is ample evidence for a role of ERK1 in growth control (Sontag et al, 1993), the diminished invasiveness produced by PD 098059 is unlikely to be due to an anti-proliferative effect for three reasons. First, the mitotic index in PD 098059-treated versus control tumours was similar. Second, we found no correlation between the size of the tumours and their invasiveness. Third, we found little evidence of diminished growth of cultured UM-SCC-1 cells (Simon et al, 1996) using concentrations of PD 098059 employed in the current study.

In our study we observed a substantial, but incomplete, attenuation of in vivo invasion and MMP-9 expression brought about by PD 098059. This might be explained in different ways. For example, the drug may be incompletely distributed throughout the tumour; a real possibility considering that the PD 098059 was injected intratumourally rather than systemically administered. However, our observation of a substantial reduction in ERK activity in PD 098059-treated tumours would argue against this possibility. It is more probable that protease expression is regulated by both PD 098059-sensitive and -insensitive signalling cascades. Indeed, we previously demonstrated that MMP-9 expression and in vitro invasion can be partially regulated via the p38 MAPK (Simon et al, 1998).

Although we showed a clear reduction in MMP-9 activity in PD 098059-treated tumours, it is unlikely that the reduced invasiveness of the tumours is entirely a consequence of the repression of this metalloproteinase. It is more probable that the repression of

*© 1999 Cancer Research Campaign* 

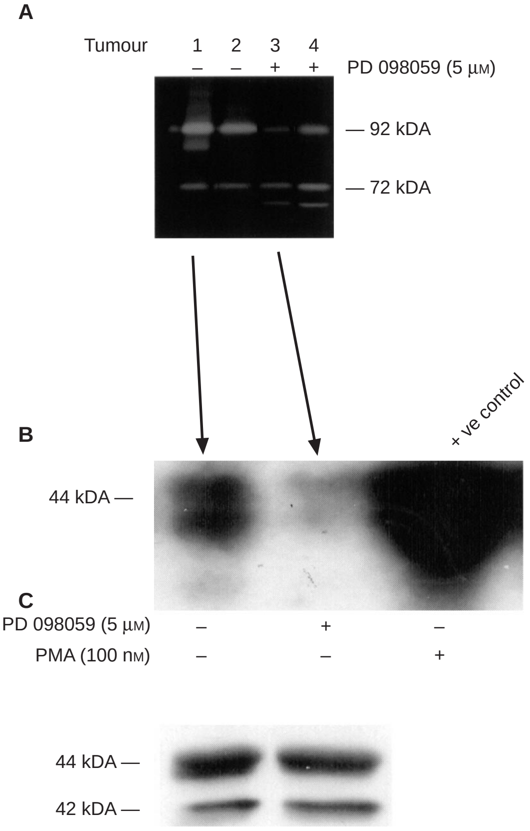

**Figure 7** Reduced MMP-9 and ERK1 activity in PD 098059-treated tumours. UM-SCC-1 cells were injected into the subcutaneous tissue superficial to the mylohyoid muscle. After 18 days, mice were treated with empty liposomes or PD 098059-liposomes. Tumours subsequently received drug/liposome injections on day 23, 28 and every third day thereafter until day 40. At that point, mice were sacrificed and tumours harvested. Tumour extracts were generated and equivalent protein amounts subjected to zymography (**A**), immunoprecipitation and an in-gel kinase assay (**B**) or Western blotting for ERK1/ERK2 (**C**). Tumours 1 and 2 were from mice treated with empty liposomes and tumours 3 and 4 were obtained from PD 098059/liposome-treated mice. The + ve control lane in Panel **B** represents extract derived from PMA-treated UM-SCC-1 cells in culture multiple AP-1-dependent proteases such as urokinase, type I collagenase, and stromelysin 2 (Sirum and Brinckerhoff, 1989; Auble and Brinckerhoff, 1991; Nerlov et al, 1991; Sato and Seiki, 1993), all of which have been implicated in the progression of head and neck cancer (Clayman et al, 1993; Muller et al, 1993), is responsible for the overall reduction in tumour invasiveness. Indeed, in a previous study, we showed that the synthesis of the u-PA in UM-SCC-1 cells was reduced by PD 098059 (Simon et al, 1996). On the other hand, we did not detect a decrease in the 72-kDa type IV collagenase (MMP-2) and this is consistent with the absence of AP-1-binding motifs in the promoter sequence (Huhtala et al, 1990).

The ability to reduce tumour cell infiltration into surrounding and adjacent tissues may ultimately be of therapeutic value for patients afflicted with oral squamous cell carcinoma where local and regional invasion represents a major challenge to the clinician. Our findings raise the exciting possibility that second-generation analogues of PD 098059 may, in an adjuvant setting, diminish the spread of residual disease following surgery/irradiation.

### ACKNOWLEDGEMENTS

This work was supported by NIH grants ROI DE10845, ROI CA58311, P50 DE11906 to D.B., a Texas Higher Education Coordinating Board Grant ATP 15091 to R.M., and a fellowship to C.S. (Si 634/1-1) from the Deutsche Forschungsgemeinschaft.

### REFERENCES

Abdel-Hafiz H, Heasley LE, Kyriakis JM, Avruch J, Kroll DJ, Johnson GL and Hoeffler JP (1996) Activating transcription factor-2 DNA-binding activity is stimulated by phosphorylation catalyzed by p42 and p54 microtubule-associated protein kinases. *Mol Endocrinol* **6**: 2079–2089

Agarwal S, Corbley MJ and Roberts TM (1995) Reconstitution of signal transduction from the membrane to the nucleus in a baculovirus expression system: activation of *raf*-1 leads to hypermodification of c-*jun* and c-*fos* via multiple pathways. *Oncogene* **11**: 427–438

Alessi DR, Cuenda A, Cohen P, Dudley DT and Saltiel AR (1995) PD 098059 is a specific inhibitor of the activation of mitogen-activated protein kinase kinase in vitro and in vivo. *J Biol Chem* **270**: 27489–27494

Anderson IC, Shipp MA, Docherty AJP and Teicher BA (1996) Combination therapy including a gelatinase inhibitor and cytotoxic agent reduces local invasion and metastasis of murine Lewis lung carcinoma. *Cancer Res* **56**: 715–718

Auble DT and Brinckerhoff CE (1991) The AP-1 sequence is necessary but not sufficient for phorbol induction of collagenase in fibroblasts. *Biochemistry* **30**: 4629–4635

Axelrod JH, Reich R and Miskin R (1989) Expression of human recombinant plasminogen activators enhances invasion and experimental metastasis of H-*ras*-transformed NIH3T3 cells. *Mol Cell Biol* **9**: 2133–2141

Bogoyevitch MA, Ketterman AJ and Sugden PH (1995) Cellular stresses differentially activate c-*jun* N-terminal protein kinases and extracellular signal-regulated protein kinases in cultured ventricular myocytes. *J Biol Chem* **270**: 29710–29717

Carter RL, Foster CS, Dinsdale EA and Pittam MR (1983) Perineural spread by squamous carcinomas of the head and neck: a morphological study using antiaxonal and antimyelin monoclonal antibodies. *J Clin Pathol* **36**: 269–275

Clayman G, Wang SW, Nicolson GL, El-Naggar A, Mazar A, Henkin J, Blasi F, Goepfert H and Boyd D (1993) Regulation of urokinase-type plasminogen activator expression in squamous cell carcinoma of the oral cavity. *Int J Cancer* **54**: 73–80

Dudley DT, Pang L, Decker SJ, Bridges A and Saltiel AR (1995) A synthetic inhibitor of the mitogen-activated protein kinase cascade. *Proc Natl Acad Sci USA* **92**: 7686–7689

Frost JA, Geppert TD, Cobb MH and Feramisco JR (1994) A requirement for extracellular signal-regulated kinase (ERK) function in the activation of AP-1 by Ha-ras, phorbol 12-myristate 13-acetate and serum. *Proc Natl Acad Sci USA* **91**: 3844–3848

Gille H, Sharrocks AD and Shaw PE (1992) Phosphorylation of transcription factor $p62^{tcf}$ by MAP kinase stimulates ternary complex formation at c-*fos* promoter. *Nature* **358**: 414–417

Goepfert H, Dichtel WJ, Medina JE, Lindberg RD and Luna MD (1984) Perineural invasion in squamous cell skin carcinoma of the head and neck. *Am J Surg* **148**: 542–547

Gum R, Lengyel E, Juarez J, Chen J, Sato H, Seiki M and Boyd D (1996) Stimulation of 92 kDa type IV collagenase promoter activity by ras is MEK1-independent and requires multiple transcription factor binding sites including closely spaced PEA3/ets and AP-1 sequences. *J Biol Chem* **271**: 10672–10682

Gum R, Wang H, Lengyel E, Juarez J and Boyd D (1997). Regulation of 92 kDa type IV collagenase expression by the *jun* aminoterminal kinase- and the extracellular signal-regulated kinase-dependent signaling cascades. *Oncogene* **14**: 1481–1493

Huang W, Alessandrini A, Crews CM and Erikson RL (1993) Raf-1 forms a stable complex with Mek1 and activates Mek1 by serine phosphorylation. *Proc Natl Acad Sci USA* **90**: 10947–10951

Huhtala P, Chow L and Tryggvason K (1990) Structure of the human type IV collagenase gene. *J Biol Chem* **265**: 11077–11082

Janknecht R (1996) Analysis of the ERK-stimulated Ets transcription factor ER81. *Mol Cell Biol* **16**: 1550–1556

Jelinek T, Catling AD, Reuter CWM, Moodie SA, Wolfman A and Weber MJ (1994)

 *© 1999 Cancer Research Campaign*

*Ras* and *raf*-1 form a signalling complex with MEK-1 but not MEK-2. *Mol Cell Biol* **14**: 8212–8218

Jensen PJ and Rodeck U (1993) Autocrine/paracrine regulation of keratinocyte urokinase plasminogen activator through the TGF-α/EGF receptor. *J Cell Physiol* **155**: 333–339

Juarez C, Clayman G, Nakajima M, Tanabe K, Saya H, Nicolson GL and Boyd D (1993) Role and regulation of 92 kDa type IV collagenase (MMP-9) in invasive squamous cell carcinoma of the oral cavity. *Int J Cancer* **54**: 73–80

Kyriakis JM, App H, Zhang X, Banerjee P, Brautigan DL, Rapp UR and Avruch J (1992) *Raf*-1 activates MAP kinase-kinase. *Nature* **358**: 417–420

Lengyel E, Wang H, Gum R, Simon C, Wang Y and Boyd D (1997) Elevated urokinase-type plasminogen activator receptor expression in a colon cancer cell line is due to a constitutively activated extracellular signal-regulated kinase-1-dependent signaling cascade. *Oncogene* **14**: 2563–2573

Lyons J, Birkedal-Hansen B, Pierson MC, Whitelock JM and Birkedal-Hansen H (1993) Interleukin-1 and TGF-α/EGF induce expression of Mr 95 000 type IV collagenase/gelatinase and interstitial fibroblast-type collagenase by rat mucosal keratinocytes. *J Biol Chem* **268**: 19143–19151

Matrisian LM (1992) The matrix-degrading metalloproteinases. *BioEssays* **14**: 455–463

Mossman T (1983) Rapid colorimetric assay for cellular growth and survival: application to proliferation and cytoxicity assays. *J Immun Methods* **65**: 55–63

Muller D, Wolf C, Abecassis J, Millon R, Engelmann A, Bronner G, Rouyer N, Rio M, Eber M, Methlin G, Chambon P and Basset P (1993) Increased stromelysin 3 gene expression is associated with increased local invasiveness in head and neck squamous cell carcinoma. *Cancer Res* **53**: 165–169

Nakajima M, Isumusa T and Nicolson GL (1992) Heparanase and tumor metastasis. *J Cell Biochem* **36**: 157–167

Nerlov C, Rorth P, Blasi F and Johnsen M (1991) Essential AP-1 and PEA3 binding elements in the human urokinase enhancer display cell type-specific activity. *Oncogene* **6**: 1583–1593

O'Malley BW, Chen S, Schwartz MR and Woo SLC (1995) Adenovirus-mediated gene therapy for human head and neck squamous cell cancer in a nude mouse model. *Cancer Res* **55**: 1080–1085

Ossowski L (1992) Invasion of connective tissue by human carcinoma cell lines: requirement for urokinase, urokinase receptor, and interstitial collagenase. *Cancer Res* **52**: 6754–6760

Ossowski L and Reich E (1983) Antibodies to plasminogen activator inhibit human tumor metastasis. *Cell* **35**: 611–619

Pepper MS, Matsumoto K, Nakamura T, Orei L and Montesano R (1992) Hepatocyte growth factor increases urokinase-type plasminogen activator (u-PA) and u-PA receptor expression in Madin–Darby canine kidney epithelial cells. *J Biol Chem* **267**: 20493–20496

Pulverer BJ, Kyriakis JM, Avruch J, Nikolakaki E and Woodgett JR (1991) Phosphorylation of c-*jun* mediated by MAP kinases. *Nature* **353**: 670–674

Rempel SA, Rosenblum ML, Mikkelsen T, Yan PS, Ellis KD, Golembieski WA, Sameni M, Rozhin J, Ziegles G and Sloane BF (1994) Cathepsin B expression and localisation in glioma progression and invasion. *Cancer Res* **54**: 6027–6031

Sato H and Seiki M (1993) Regulatory mechanism of 92 kDa type IV collagenase gene expression which is associated with invasiveness of tumor cells. *Oncogene* **8**: 395–405

Simon C, Juarez J, Nicolson GL and Boyd D (1996) Effect of PD 098059, a specific inhibitor of mitogen-activated protein kinase kinase, on urokinase expression and in vitro invasion. *Cancer Res* **56**: 5369–5374

Simon C, Goepfert H and Boyd D (1998). Inhibition of the p38 mitogen-activated protein kinase by SB 202580 blocks PMA-induced Mr 92 000 type IV collagenase secretion and in vitro invasion. *Cancer Res* **58**: 1135–1139

Sirum KL and Brinckerhoff CE (1989) Cloning of the genes for human stromelysin and stromelysin 2: differential expression in rheumatoid synovial fibroblasts. *Biochemistry* **28**: 8691–8698

Sontag E, Dedorov S, Kamibayashi C, Robbins D, Cobb M and Mumby M (1993) The interaction of SV40 small tumor antigen with protein phosphatase 2A stimulates the MAP kinase pathway and induces cell proliferation. *Cell* **75**: 887–897

Tryggvason K, Hoyhtya M and Salo T (1987) Proteolytic degradation of extracellular matrix in tumor invasion. *Biochim Biophys Acta* **907**: 191–217

Wilhelm SM, Collier IE, Marmer BL, Eisen AZ, Grant G and Goldberg G (1989) SV40-transformed human lung fibroblasts secrete a 92-kDa type IV collagenase which is identical to that secreted by normal human macrophages. *J Biol Chem* **264**: 17213–17221

Wilhelm O, Schmitt M, Hohl S, Senekowitsch R and Graeff H (1995) Antisense inhibition of urokinase reduces spread of human ovarian cancer in mice. *Clin Exp Metastasis* **13**: 296–302

Xing RH, Mazar A, Henkin J and Rabbani SA (1997) Prevention of breast cancer growth, invasion and metastasis by antiestrogen tamoxifen alone or in combination with urokinase inhibitor B-428. *Cancer Res* **57**: 3585–3593

Zheng C and Guan K (1994) Activation of MEK family kinases requires phosphorylation of two conserved Ser/Try residues. *EMBO J* **13**: 1123–1131

Ziegler G and Sloane BF (1998) Cathepsin B expression and localization in glioma progression and invasion. *Cancer Res* **54**: 6027–6031

*© 1999 Cancer Research Campaign*
